# ZTE MRI for Rotator Cuff Tear Arthropathy: Integrated Bone–Muscle Analysis and Its Association with Pseudoparesis

**DOI:** 10.3390/jcm14238597

**Published:** 2025-12-04

**Authors:** Engin Türkay Yılmaz, Serkan İbik, Vedat Yaman, Şeyda Betül Fındık, Üstün Aydıngöz, Gazi Huri

**Affiliations:** 1 Department of Orthopedics and Traumatology, Alaca State Hospital, 19600 Corum, Türkiye; 2 Department of Orthopedics and Traumatology, Hacettepe University Hospitals, 06230 Ankara, Türkiye; serkanibik@gmail.com (S.İ.); gazihuri@yahoo.com (G.H.); 3 Department of Radiology, Hacettepe University Hospitals, 06230 Ankara, Türkiye; drvedatyaman@gmail.com (V.Y.); ustunaydingoz@yahoo.com (Ü.A.); 4 Emergency Health Services Department, Ankara Provincial Health Directorate, 06510 Ankara, Türkiye; seydabetulfindik@gmail.com; 5 Department of Radiology, SSM Saint Louis University Hospital, St. Louis, MO 63104, USA; 6 Department of Orthopaedic and Sports Medicine, Hospital Doha, Doha P.O. Box 9958, Qatar

**Keywords:** magnetic resonance imaging, zero echo time imaging, cuff tear arthropathy, pseudoparesis, shoulder

## Abstract

**Background/Objectives:** Evaluating glenoid changes in rotator cuff tear arthropathy (RCTA) is crucial for preoperative planning. MRI with zero echo time (ZTE) sequence, which produces CT-like images, allows for the assessment of osseous morphology as well as factors contributing to pseudoparesis in RCTA patients. **Methods:** In this retrospective study, using 3T MRI, glenoid version, glenoid vault depth, humeral subluxation index, humeral head medialization, critical shoulder angle, glenoid best-fit circle width, glenoid best-fit circle bone loss ratio (GBLR), and anterior, central, and posterior glenoid bone loss were measured on reformatted 3D ZTE images in 43 shoulders independently by three observers. The same measurements were repeated by one observer after 10 days. Muscle cross-sectional areas were measured. Patients’ active ROMs, American Shoulder and Elbow Surgeons (ASES), and Constant–Murley scores were recorded. Patients unable to perform 90° forward elevation were classified as the pseudoparesis group. **Results:** Interobserver agreements were good to excellent, except for glenoid vault depth, anterior bone loss, and GBLR. Intraobserver agreements were good to excellent. The pseudoparesis group showed significantly less subscapularis muscle cross-sectional area (*p* = 0.006). Moderate correlations were found between subscapularis cross-sectional area and forward elevation, abduction, and internal rotation ([r = 0.471, *p* = 0.001]; [r = 0.447, *p* = 0.003]; [r = 0.464, *p* = 0.002], respectively). Moderate negative correlations were found between anterior glenoid loss and forward elevation (r = −0.411, *p* = 0.006) and abduction (r = −0.475, *p* = 0.001). **Conclusions:** MRI with ZTE sequence demonstrated good reliability for assessing osseous morphology in shoulders with RCTA. Glenoid anterior bone loss and loss of subscapularis muscle are both associated with pseudoparesis.

## 1. Introduction

Rotator cuff tear arthropathy (RCTA) is a common shoulder problem in the elderly population characterized by the triad of massive rotator cuff tears, glenohumeral joint osteoarthritis, and superior migration of the humerus [1,2]. Computed tomography (CT) is the current standard imaging method for the preoperative evaluation of patients, facilitating the selection of surgical technique, implant selection, and outcome assessment [3,4,5]. On the other hand, Magnetic resonance imaging (MRI) enables the estimation of muscle stock and quality while helping to visualize other critical soft tissue structures such as tendons, nerves, and joint capsules [6,7,8]. Patients with RCTA usually undergo MRI first, which establishes the diagnosis and provides guidance in determining surgery, but a CT may be required for precise assessment of bony structures that are relevant in surgical planning.

Zero echo time (ZTE, zero time to echo) sequence is a recent MRI technique that picks up scant cortical bone signal, which is lost in conventional MRI sequences, thereby generating CT-like images with a silent 5–7 min acquisition [9]. ZTE MRI has already been validated with CT in certain shoulder-related orthopedic settings, such as glenohumeral osteoarthritis, fractures, anterior shoulder instability, and critical shoulder angle measurement [10,11,12,13,14,15]. Additionally, in recent years, ZTE MRI has been validated against CT in other areas of orthopedics as well, including pediatric sacroiliitis, ankylosing spondylitis, and osteolytic lesions in multiple myeloma [16,17,18].

Preoperative functional assessment of patients with an already established RCTA is also pivotal in determining the type of surgery and informing patient expectations following surgery. In addition to glenohumeral joint deformity due to chronic rotator cuff tears, the development of pseudoparesis and pseudoparalysis further contributes to functional impairment in patients with RCTA. In pseudoparalysis and pseudoparesis, there are active motion deficits in abduction, forward elevation, and external and internal rotations [19]. Pseudoparalysis has been historically defined as a limitation in active shoulder movements due to rotator cuff tear without neurological impairment [20]. Pseudoparesis was defined by Werner as the presence of free passive anterior elevation, inability to actively elevate the arm beyond 90°, and the absence of neurological deficits [21]. Although there is no definitive consensus in the literature, this definition is mostly used [22]. Biomechanical and clinical studies have suggested that pseudoparesis can be summarized as a consequence of disrupted humeral head centralization on the glenoid, leading to an imbalance of anterior and posterior force couples and impaired deltoid elevation function [23]. Several studies have particularly indicated that the condition of the subscapularis muscle is predictive of pseudoparesis [24,25]. To our knowledge, this appears to be the first study to concurrently evaluate glenoid morphology using ZTE MRI and assess rotator cuff muscle cross-sectional areas through preoperative cross-sectional imaging while also examining their relationship with shoulder movement in patients with RCTA. This integrated approach may offer a novel perspective for surgical planning and outcome assessment.

In this hybrid study evaluating both the imaging technique and clinical outcomes, we aimed to establish the reliability of osseous measurements on ZTE MRI in addition to conventional shoulder MRI in the preoperative work-up of RCTA and to investigate how these measurements and muscle cross-sectional areas relate to functional outcomes.

## 2. Materials and Methods

This single-center retrospective study was approved by Hacettepe University Ethics Committee (protocol code GO:23/136, 4 April 2023), and the informed consent requirement was waived. This study was prepared in accordance with the Strengthening the Reporting of Observational Studies in Epidemiology guidelines for reporting observational studies in epidemiology [26]. Informed consent was acquired from all the subjects who participated in the study. In addition, information was provided to the patients explaining that data from their cases would be submitted for publication, and their consent was provided.

All patients aged 50 years and above, who had American Shoulder and Elbow Surgeons (ASES) and Constant–Murley scores along with range of motion (ROM) measurements noted during the orthopedic outpatient clinic and underwent 3T MRI at our institution between July 2021 and January 2023, formed the initial study population. The inclusion criterion was the MRI-based diagnosis of RCTA. Exclusion criteria were rheumatologic diseases, soft tissue and bone malignancies, radiotherapy to the shoulder, previous fractures of the proximal humerus and scapula, and patients with frozen shoulder, cervical stenosis, and brachial plexus injury causing restriction in shoulder movements and weakness in shoulder girdle muscles.

- MRI examinations

All shoulder MRI evaluations in this research were carried out on a 3 Tesla MRI unit (Signa Architect; GE HealthCare, Chicago, IL, USA) with a dedicated surface coil. The standard MRI protocol included an isotropic (i.e., 1 × 1 × 1 mm voxel size) coronal 3D ZTE sequence in addition to the sagittal oblique T1-weighted (T1W), along with fat-saturated T2-weighted (T2W), coronal oblique and axial fat-saturated T2W, and axial proton density-weighted (PDW) sequences. Patients’ hands were in a supine position, and the palm of the same-sided hand faced the body while imaging was performed.

- Training and measurement sessions

A trainer (Ü.A.) who had 27 years of experience in musculoskeletal radiology provided training on the generation of ZTE sequence-based image reformations for a fifth-year orthopedics resident (E.T.Y.; Observer 1) using five people with healthy shoulders (who were outside the study group) as subjects. During the training session, which entailed using the picture archiving and communications system (PACS) and creating reformatted images, technical steps were replicated under the trainer’s supervision. The resident then independently created image reformations on the first, third, and fifth days post-training, seeking the trainer’s guidance for any difficulties. Following a one-week break, the resident repeated this independent three-day training sequence. After the creation of reformatted images, image validation was made by the trainer. Training on measurement methods was then given by the trainer to a fourth-year radiology resident (V.Y; Observer 2) and a first-year orthopedic resident (S.İ; Observer 3) alongside the initial observer.

The initial measurements were made by all the observers for interobserver reliability. To evaluate intraobserver reliability, Observer 1 repeated the measurements after a 10-day hiatus. Observers were blinded to patient clinical information and to each other’s measurements to minimize potential bias.

- MRI-based measurements

**Figure 1:**
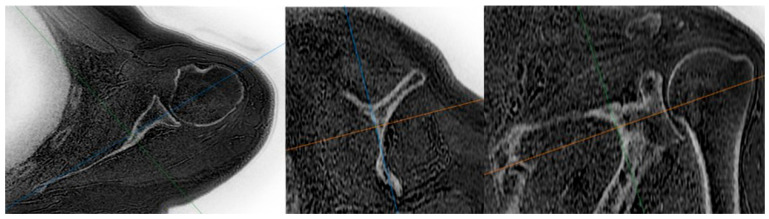
The true scapular plane was established as being parallel to the scapular body in sagittal sections, passing from the center of the glenoid to the trigonum scapulae in the coronal plane and extending from the center of the glenoid to the medial border of the scapula in the transverse plane. The axial, sagittal, and coronal reference planes are represented by the orange, green and blue lines, respectively.

**Figure 2:**
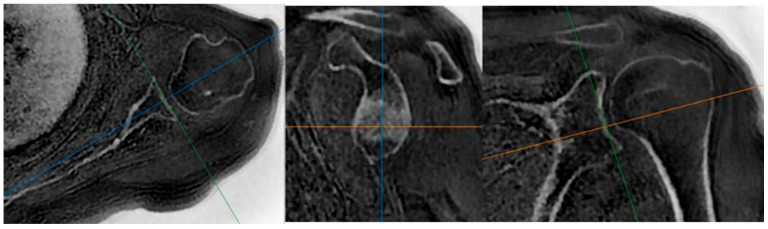
The glenoid en face view was obtained by reconstructing the image parallel to the scapular body in the sagittal plane, aligning it parallel to the surface of the inferior two-thirds of the glenoid articular face in the coronal plane and orienting it such that the longest axis of the pear-shaped glenoid was directed anteroinferiorly. The axial, sagittal, and coronal reference planes are represented by the orange, green and blue lines, respectively.

A web-based workstation (AW software, version 2.3, GE HealthCare, Chicago, IL, USA) program was used to create the true scapular plane from the obtained ZTE images, enabling accurate measurements on axial and sagittal sections. Additionally, black-and-white raw images were inverted to obtain CT-like images (Figure 1) [27]. To display the glenoid surface in an en face view, another image was produced to view the surface aligned with the lower one-third of the glenoid surface (Figure 2) [13].

**Figure 3:**
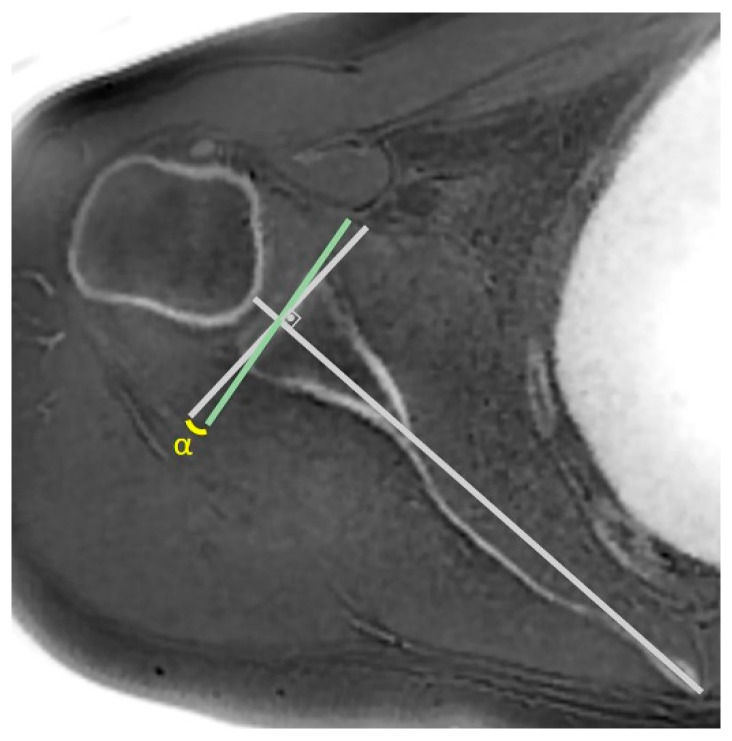
Glenoid version was measured on 2D ‘true scapular plane’ reformats generated from the 3D-reconstructed ZTE MRI images using the Friedman method. A line was drawn from the medial border of the scapula (medial tip of the scapular spine) to the midpoint of the glenoid cavity (the Friedman line). The angle between a line perpendicular to the Friedman line and a line connecting the anterior and posterior rims of the glenoid was defined as the glenoid version.

**Figure 4:**
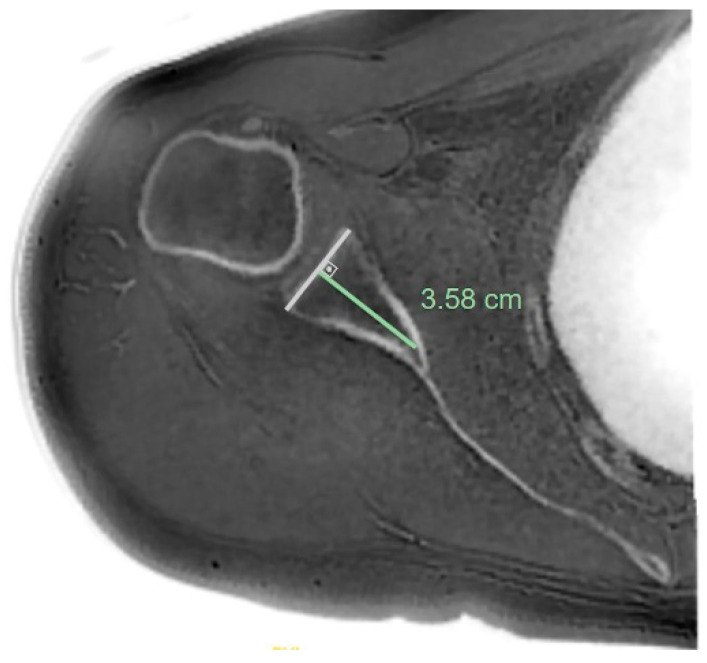
Glenoid vault depth was measured on true scapular plane reformat images by first drawing a line connecting the anterior and posterior margins of the glenoid. From the midpoint of this line, a perpendicular line was drawn toward the deepest point of the glenoid endosteal wall.

**Figure 5:**
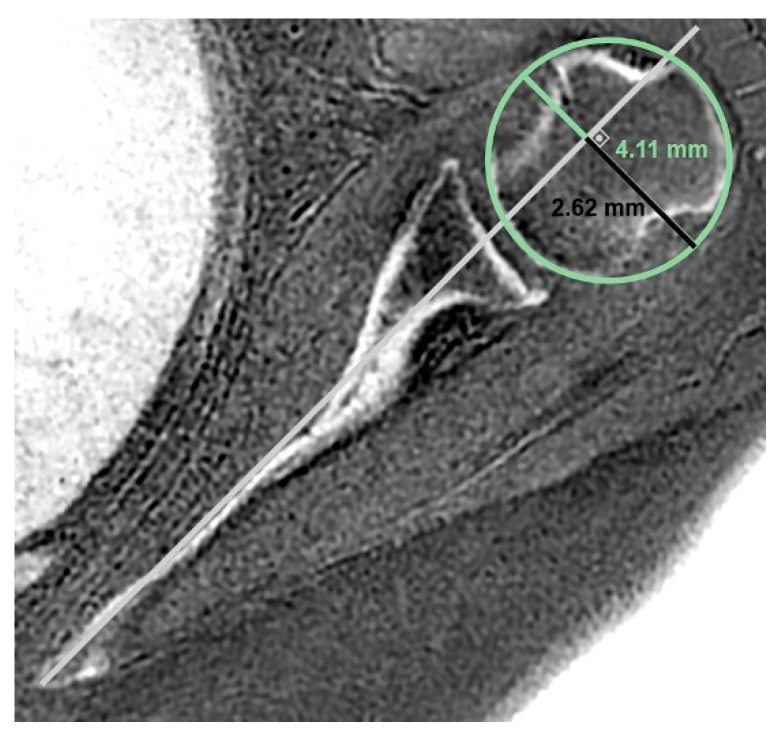
The humeral head subluxation index (HSI) was measured on a true scapular plane reformat by first placing a best-fit circle to the articular surface of the humeral head. The longest perpendicular distance from the posterior edge of the best-fit circle to the Friedman line was measured. The ratio of this distance to the diameter of the circle was calculated as the HSI.

**Figure 6:**
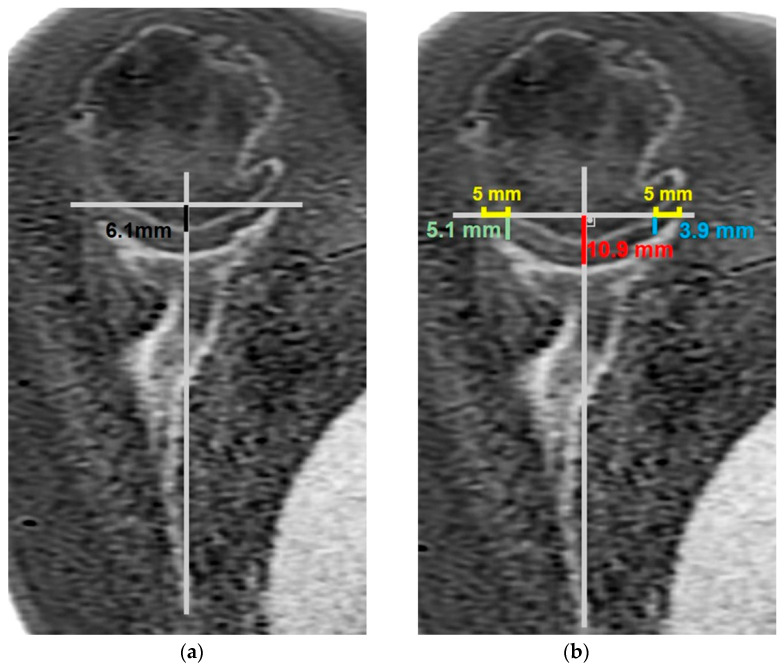
(**a**) Measurement of humeral head medialization. This was measured by first drawing a line perpendicular to the Friedman line from the most lateral point of the glenoid. The shortest distance from this perpendicular line to the most medial point of the humeral head (if located medial to this line) was defined as the medialization distance. (**b**) Measurement of anterior (blue line), central (red line), and posterior (green line) bone loss. The bone loss was assessed by measuring the shortest distances from the central point of the glenoid, a point located 5 mm posterior to the anterior glenoid corner, and a point located 5 mm anterior to the posterior glenoid corner to the same perpendicular reference line.

In the true scapular plane, glenoid version measurements were made according to the Friedman method (Figure 3), glenoid vault depth (Figure 4), humeral subluxation index (HSI) (Figure 5), and humeral head medialization, and anterior, central, and posterior glenoid bone loss were measured (Figure 6) [28,29].

**Figure 7:**
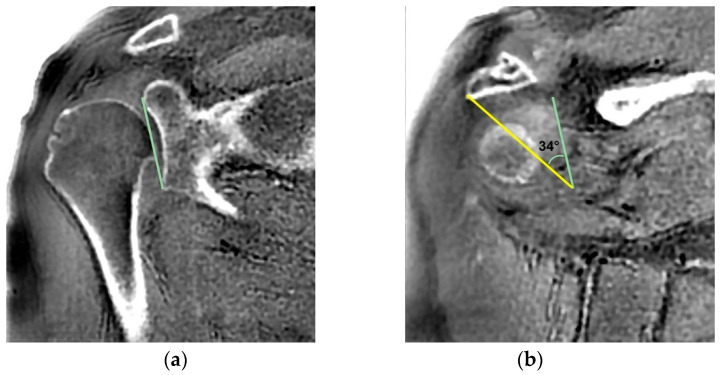
CSA was measured on coronal reformats aligned with the true scapular plane. (**a**) On the section where the superior and inferior margins of the glenoid were visible, a line was drawn connecting these two points. (**b**) On the section where the most lateral point of the acromion was visible, a second line was drawn from this point to the most inferior point of the previously drawn glenoid line. The angle formed between these two lines was defined as the CSA.

**Figure 8:**
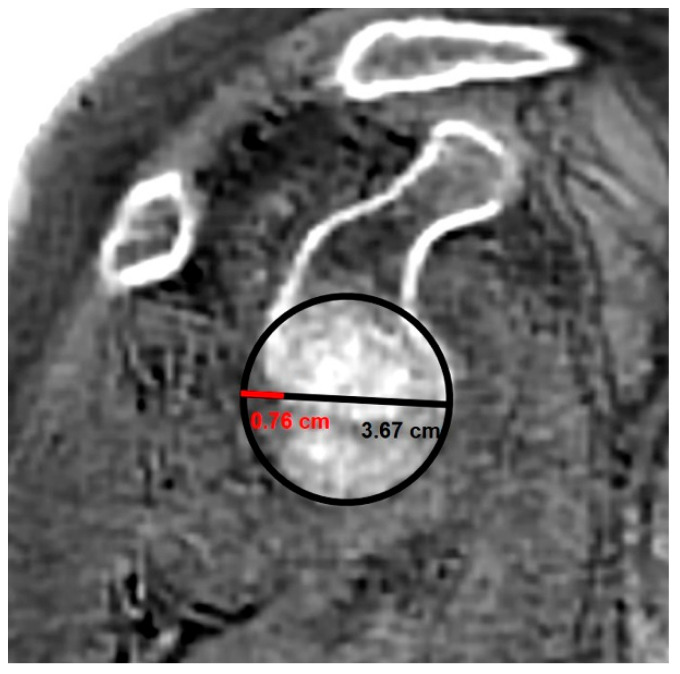
A glenoid best-fit circle was created by drawing a circle on the en face view that passed through the inferior and anterior margins of the glenoid to ensure optimal fitting in the slice where bone loss is most clearly visualized. The extent of posterior bone loss was measured as the distance between the posterior margin of the glenoid and the posterior border of the circle (red line). This distance was then divided by the diameter of the circle to calculate the glenoid best-fit circle bone loss ratio (GBLR).

**Figure 9:**
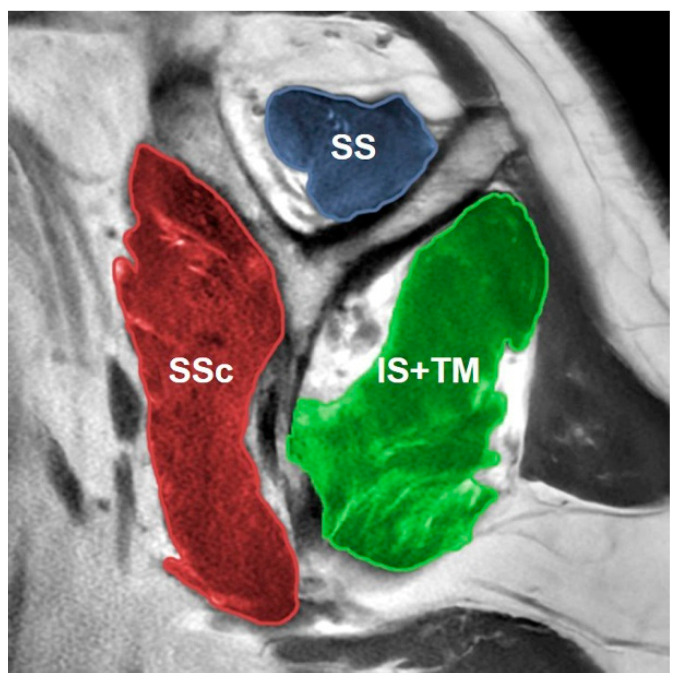
On sagittal T1-weighted MRI, at the level where the ‘Y-scapula’ appearance was most lateral, the cross-sectional areas of the subscapularis (SSc) and supraspinatus (SS) muscles were measured individually, whereas the infraspinatus (IS) and teres minor (TM) muscles were measured together as a single muscle group.

CSA was measured on coronal reformats aligned with the true scapular plane, derived from the ZTE MRI reconstruction (Figure 7). A glenoid best-fit circle was made by drawing a circle on the en face plane that passed through the anterior and inferior aspects of the glenoid, ensuring a perfect fit (Figure 8). The length of bone loss in the posterior region was divided by the full diameter of the circle to determine the glenoid best-fit circle bone loss ratio (GBLR). On sagittal T1W MRI, at the level where the ‘Y-scapula’ appearance was observed most laterally, the cross-sectional areas of the subspinatus (SS) and subscapularis (SSc) muscles were measured individually, while the teres minor (TM) and infraspinatus (IS) muscles were measured together as a single muscle group (Figure 9). The SSc/IS + TM ratio was calculated to assess the ratio of shoulder internal rotation to external rotation forces. Patients with shoulder forward elevation measurements < 90° were categorized into the pseudoparesis group, while those with measurements of ≥90° were classified into the nonparesis group.

- Statistical analysis

SPSS version 26.0 (SPSS Inc., Chicago, IL, USA) was used for statistical analysis. Normal distribution of quantitative data was assessed using the Shapiro–Wilk test and Kolmogorov–Smirnov test, while variance homogeneity was checked using Levene’s test. In assessing interobserver and intraobserver agreement, the intraclass correlation coefficient (ICC) was determined by a two-way mixed-effect model through single measurement and absolute-agreement, along with a confidence interval (CI) of 95%. ICC values above 0.90 were considered excellent, between 0.75 and 0.89 were good, between 0.5 and 0.74 were moderate, and below 0.5 were considered poor reliability [30]. For parametric values, the Pearson correlation test was used, and for nonparametric values, the Spearman correlation test was used to assess the relationship between ZTE bone measurements, T1W MRI muscle cross-sectional areas, ASES and Constant–Murley Scores, and shoulder joint motion ranges. For parametric data, group differences were evaluated using the *t*-test, whereas the Mann–Whitney U test was applied for nonparametric data. Parametric variables were summarized using mean and standard deviation, whereas nonparametric variables were summarized using median and interquartile range (IQR), with the IQR defined as the difference between the 25th and 75th percentiles to reflect data dispersion. Correlation coefficients between 0 and 0.09 were considered negligible correlation, between 0.10 and 0.39 were weak correlation, between 0.40 and 0.69 were moderate correlation, between 0.70 and 0.89 were strong correlation, and between 0.90 and 1 were very strong correlation [31]. A threshold of *p* less than 0.05 was used to determine statistical significance across analyses.

- Power analysis

For correlation analyses, the sample size was calculated using G*Power 3.1 (Heinrich Heine University, Düsseldorf, Germany) based on data from a previous study by Siebert et al. [28]. To detect a correlation coefficient of 0.511 between two variables, with a significance level of 0.05 (Type I error) and 90% power, at least 36 patients were required. For ICC analyses, the sample size was determined using PASS 15.0 (NCSS, LLC, Kaysville, UT, USA). To detect an ICC of 0.70 among three observers, with a significance level of 0.05 and 95% power, at least 26 patients were needed. Similarly, for Kappa coefficient assessment between two observers, a sample of at least 26 patients was required to detect a Kappa of 0.70 with 5% significance and 95% power.

## 3. Results

**Figure 10:**
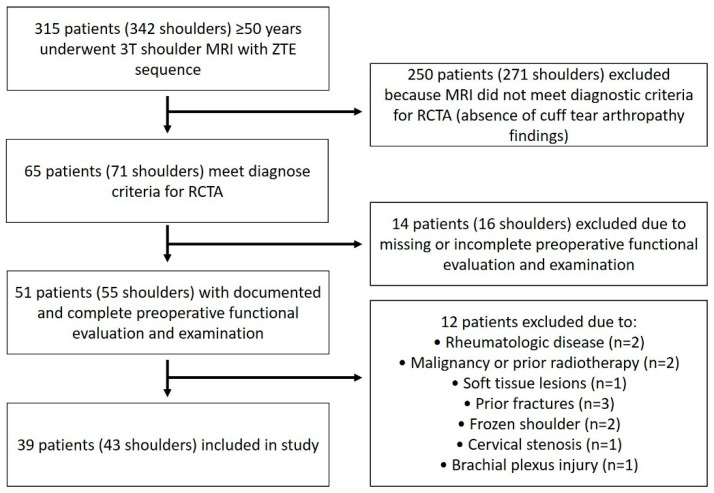
Flowchart demonstrating patient selection and exclusion process for the study cohort.

Of the 315 patients (342 shoulders) that belonged to the initial study population, 39 (43 shoulders) formed the final study population per inclusion and exclusion criteria (Figure 10). Participants presented a mean age of 70.71 ± 1.14 years. The study comprised 28 (71.8%) females and 11 (28.2%) males.

For measurements of osseous morphology, interobserver agreement was good to excellent, excluding glenoid vault depth, anterior bone loss, and best-fit circle loss ratio. In all intraobserver measurements, there was good to excellent agreement (Table 1).

The correlation between the cross-sectional area of the SSc muscle and Constant–Murley score, as measured on T1W MRI, was found to be r = 0.495 (*p* = 0.001), and with the ASES score, it was r = 0.460 (*p* = 0.002). No significant association was observed between the cross-sectional areas of the SS, IS, and TM muscles, or the SSc/IS + TM ratio and either ASES or Constant–Murley scores (Table 2). The cross-sectional area of the SSc muscle showed moderate correlations with anterior elevation, abduction, and internal rotation (r = 0.471, r = 0.447, and r = 0.464, respectively, *p* < 0.05). There was no significant correlation between active joint motion range and cross-sectional area of SS, IS, and TM.

Anterior glenoid bone loss showed a weak negative correlation with Constant–Murley score and ASES score (r:−0.322, *p* = 0.035 and r: −0.327, *p* = 0.032, respectively). No other significant correlations were found between other bone morphological changes and functional outcomes (Table 2). A moderate negative correlation was identified between anterior glenoid loss and forward elevation and abduction (r = −0.411, *p* = 0.006 and r = −0.475, *p* = 0.001, respectively). Humeral head medialization and central and posterior glenoid bone loss showed weak negative correlation with abduction (r = −0.314, *p* = 0.040; r = −0.354, *p* = 0.020; and r = −0.354, *p* = 0.020, respectively).

A comparison of bony measurements and muscle cross-sectional areas in the pseudoparesis and nonparesis groups revealed a significant difference in the SSc muscle cross-sectional area (Table 3). Furthermore, although the Constant–Murley (32.85 ± 10.53 vs. 55.75 ± 6.76, *p* = 0.154) and ASES scores (34.22 ± 7.96 vs. 57.12 ± 7.12, *p* = 0.606) did not differ significantly between the two groups, statistically meaningful differences were identified in forward elevation (median: 70, IQR: 45–70 vs. median: 100, IQR:96.25–108.75), abduction (median: 50, IQR: 45–70 vs. median: 100, IQR:95–103.75), external rotation (median: 55, IQR: 45–65 vs. median: 75, IQR:70–78.75), and internal rotation (median: 50, IQR: 30–55 vs. median: 64.5, IQR: 60–65).

## 4. Discussion

Among the results, two findings stand out as most significant. MRI with a ZTE sequence is a reliable method for effective evaluation of the bone morphology, together with the assessment of muscle stock, and the decrease in the cross-sectional area of the SSc muscle and anterior glenoid bone loss contribute to pseudoparesis in patients with RCTA. The SSc muscle cross-sectional area was significantly reduced in the pseudoparesis group (*p* = 0.007). Interobserver agreement was found to vary between moderate and excellent. Excellent interobserver agreements were found in angular measurements, such as glenoid version and CSA.

The reduction in the SSc muscle cross-sectional area was moderately correlated with active elevation, internal rotation, abduction, and Constant–Murley and ASES scores. In a study by Ernstbrunner et al., which evaluated patients with 50 massive cuff tears, the study population was split into pseudoparesis and pseudoparalysis groups according to their abduction range [32]. In the pseudoparesis group with abduction between 45° and 90°, the mean Goutallier stage was 1.6, while it was 2.9 in the pseudoparalysis group, which cannot perform an abduction above 45° (*p* < 0.001). In the pseudoparalysis group, anterior tears generally involved more than half of the SSc muscle. Wieser et al. evaluated 20 patients with massive cuff tears, and no significant difference was found in SS, IS, and TM fatty infiltration and posterior extension of the tear between patients with and without pseudoparalysis [25]. There was a notable difference between the extent of global tear and the fatty infiltration of the SSc muscle. In patients with full-thickness SS and IS tears, extension of the tear into the inferior portion of the SSc was associated with an inability to actively abduct the arm beyond 90°. In a recent study by Kanatlı et al., no association was found between pseudoparesis and subscapularis fatty degeneration. Patients with more extensive subscapularis injuries, particularly those affecting the tendon, were more prone to experience pseudoparesis [33]. Kawano et al. examined passive pendular motion of the glenohumeral joint under various rotator cuff conditions using nine cadaveric specimens [34]. They found that when both SS and SSc are insufficient, there was the highest radius of humeral translation, indicating that the SSc muscle plays a significant role in maintaining the humeral head in a central position. In the study of Kim et al., they investigated 153 shoulders with posterosuperior cuff tears and an intact subscapularis tendon. Multivariable logistic regression revealed several independent predictors of subscapularis muscle atrophy and fatty infiltration, including female sex (OR 5.6; 95% CI 1.7–18.6), increasing age (OR 1.1; 95% CI 1.0–1.1), higher supraspinatus Goutallier grade (OR 3.2; 95% CI 1.5–6.9), and the presence of synovitis (OR 2.8; 95% CI 1.1–7.9). Importantly, the patients with subscapularis atrophy and fatty infiltration exhibited reduced forward flexion and lower ASES and Constant–Murley scores, suggesting that diminished subscapularis muscle quality can adversely influence shoulder function even when the tendon itself remains structurally intact [35]. Based on the findings of this study, the addition of ZTE to conventional MRI may help evaluate subscapularis integrity, a parameter that has been linked to, but not demonstrably causative of, differences in clinical outcomes in previous work.

For bone morphological measurements in our study, only anterior glenoid bone loss showed a moderately significant negative correlation with active abduction and forward elevation (r = −0.475, *p* = 0.001; and r = −0.411, *p* = 0.006, respectively). Although there were no significant correlations between humeral medialization, central and posterior glenoid bone loss, and active elevation, these variables demonstrated a weak negative correlation with active abduction. It has been proposed that the compressive force exerted by the shoulder muscles on the humeral head during contraction is crucial for shoulder stability [36]. Taken together, these findings indicate that glenoid bone loss and reduced subscapularis strength may be associated with poorer shoulder function, potentially reflecting alterations in the force-coupling mechanism. However, given the retrospective design, this relationship should be interpreted cautiously, and future biomechanical studies are needed to clarify the nature of this association.

In the study by Moor et al., which evaluated 298 shoulders, significantly higher CSA values were observed in shoulders with degenerative rotator cuff tears [37]. Lu et al. found that patients with RCTA who were able to perform active forward elevation above 90 degrees had significantly lower mean CSA values [38]. In another retrospective study evaluating true anteroposterior radiographs of 1000 patients, rotator cuff-teared shoulder mean CSA values were highest, whereas lower CSA values were found in patients with osteoarthritis [39]. In contrast, our study did not reveal any significant correlation between CSA values and functional outcomes. A more comprehensive understanding of how an increased CSA, which results from a more lateralized acromion and a superiorly inclined glenoid, contributes to the development of RCTA and affects functional outcomes could yield important perspectives for both clinical practice and subsequent studies.

In the shoulder literature, several studies utilizing ZTE MRI have been published in recent years [10,11,12,13,14,15,40,41]. In the study by De Mello et al., glenoid width was measured in an en face view using ZTE MRI and CT for comparison. The analysis included six shoulder cadavers examined both before and after creating a glenoid defect, as well as ten patients with shoulder instability [11]. It was demonstrated that the ICC coefficients between modalities and interobserver agreement were excellent in both patients and cadavers. In the validation study by Breighner et al., which compared ZTE MRI with CT in the evaluation of 34 shoulders, the agreement between the two modalities yielded an ICC of 0.71 and 0.84 for glenoid vault depth and 0.98 and 0.95 for glenoid version for two observers [10]. In the study by Oishi et al., which assessed glenoid bone loss in patients with anterior shoulder instability using the best-fit circle method on en face images with both 3D CT and 3D ZTE MRI, the correlation between the two modalities was reported as a Spearman’s rank correlation coefficient of 0.89 (95% confidence interval [CI], 0.85–0.96; power = 1.00) [15]. In another study evaluating 65 shoulders, the intermodality agreement of CSA between true anteroposterior radiographs and ZTE MRI was found to be moderate (ICC = 0.66; 95% CI, 0.48–0.73) [12]. In this study, the reliability of HSI, humeral head medialization, anterior, central, and posterior bone loss, as well as GBLR, was evaluated for the first time in ZTE MRI. In our study, interobserver agreement for angular measurements was excellent, with an ICC of 0.906 (95% CI, 0.811–0.951) for glenoid version and 0.945 (95% CI, 0.845–0.947) for CSA. Lower reliability was observed for smaller-scale measurements, such as anterior glenoid bone loss and GBLR. The moderate agreement achieved in glenoid vault depth measurement may be attributed to the difficulty in distinguishing trabecular bone from cortical bone on ZTE MRI. In a recent study of patients with anterior shoulder instability, ZTE MRI data were reconstructed using both standard and deep learning techniques. The deep learning reconstructions provided markedly improved perceived resolution (OR = 7.67, *p* = 0.01) and substantially enhanced visualization of glenoid lesions (OR = 25.12, *p* = 0.01). Importantly, for this instability population, key quantitative metrics, such as the glenoid track and Hill–Sachs interval, demonstrated nearly perfect concordance between deep learning-optimized ZTE MRI and CT, with an overall ICC of 0.99 (95% CI, 0.97–0.99) [14]. In a recent investigation, supplementing routine MRI with ZTE yielded notable gains in sensitivity for identifying calcific deposits—from 0.57 (95% CI: 0.44–0.70) to 0.77 (95% CI: 0.65–0.87) for one reader and 0.48 (95% CI: 0.35–0.61) to 0.75 (95% CI: 0.63–0.86) for the other—yet overall performance remained below that of radiography. Still, the considerable incidence of soft tissue hyperintensity artifacts on ZTE raises concerns regarding its consistency and clinical applicability [40]. Although CT remains the standard modality for preoperative planning in cuff tear arthropathy, ZTE MRI offers the unique benefit of simultaneously assessing both osseous morphology and muscle quality without exposing patients to radiation. Despite MRI generally being more costly and time-consuming than CT, obtaining comprehensive bone and muscle information in a single examination may, in selected cases, lessen the need for an additional CT study. Nonetheless, larger comparative investigations are required before ZTE MRI can be regarded as a viable alternative to CT.

In our study, ZTE MRI measurements could not be directly compared with CT-based measurements; however, they can be interpreted in the context of previous CT studies evaluating the same glenoid parameters. In our cohort, glenoid version demonstrated excellent intraobserver and interobserver agreement. In the literature, while several studies using the Friedman method have also reported excellent reproducibility, there are others showing only moderate intraobserver and interobserver reliability. For glenoid vault depth, studies using different CT-based measurement techniques have reported wide variability in interobserver agreement, ranging from 0.19 (95% CI, 0.081–0.287) to 0.58 (95% CI, 0.35–0.75) and 0.866 (95% CI, 0.825–0.899) [28,42,43]. In our study, intraobserver reliability was excellent; however, interobserver reliability was moderate, with an ICC of 0.705 (95% CI, 0.497–0.833), demonstrating considerable variability. This may be attributed either to the technical challenges of assessing the glenoid vault using ZTE MRI or to the inherently low reproducibility of this measurement method, as suggested by the broad range of CT-based reliability values reported in the literature. While interobserver agreement was good for central and posterior bone loss, it was only moderate for anterior bone loss, with an ICC of 0.67 (95% CI, 0.235–0.876). In the CT-based study by Siebert et al., interobserver agreement for anterior bone loss was reported as 0.78 (95% CI, 0.61–0.87). In the same study, posterior bone loss demonstrated an ICC of 0.76 (95% CI, 0.59–0.86), which is comparable to our findings, whereas central bone loss showed a lower agreement of 0.69 (95% CI, 0.50–0.82) relative to our results [28]. These findings suggest that bone loss measurements obtained with ZTE MRI yield results generally consistent with those reported using CT. However, the lower reliability observed for anterior bone loss—which, in our study, showed the strongest correlation with shoulder function—indicates that this association should be interpreted with caution, as the measurement variability may limit the robustness of this finding. In the CT literature, interobserver reliability for CSA measurements has been reported to be as high as 0.943 and 0.989 [44,45]. In our study as well, we observed excellent interrater reliability for CSA. Taken together, these findings suggest that CSA measurements obtained using ZTE MRI may yield results comparable to those acquired with CT. ICC values obtained in our study were comparable to ICC values reported in the CT literature (Table 4).

This study has several limitations. The number of patients was limited due to the traditional employment of CT, rather than MRI, in the routine preoperative assessment of RCTA patients. Moreover, no concurrent CT was performed on the patients since the images were retrospectively evaluated; therefore, the intermodality agreement between ZTE MRI and CT could not be assessed. However, ZTE MRI has already been validated for its accurate depiction of osseous structures in comparison to CT in the shoulder and elsewhere in the body [10,11,56,57,58]. Some key imaging features, particularly measurements of glenoid bone loss, demonstrated relatively low interobserver agreement. Given that anterior, central, and posterior bone loss are negatively correlated with functional outcomes, including pseudoparesis, the limited reliability of this metric should be acknowledged. Future studies should aim to refine measurement protocols or establish standardized criteria to improve consistency, as variability in this parameter may influence the clinical interpretation of the imaging results. The study cohort consisted entirely of participants aged 50 years or older, thus reducing the external validity of our results. Physiological characteristics such as bone density and muscle atrophy patterns may differ in younger populations, particularly in women; our results should be interpreted as primarily applicable to the elderly population. Future studies including younger cohorts are warranted to validate the broader applicability of these findings. Overall, the small sample size, absence of a CT reference, and inconsistent bone loss measurements weaken the strength of the conclusions that can be drawn and should be taken into account when interpreting the findings. In this context, ZTE MRI is not intended to replace CT but rather to complement conventional MRI by enabling concurrent assessment of bone morphology and muscle status within a single, radiation-free examination. The ZTE sequence can be integrated into a standard MRI protocol in approximately five minutes, supporting its practical feasibility as an adjunct technique in selected cases and potentially providing additional information in younger patients that may influence surgical decision-making toward anatomic rather than reverse procedures. Moreover, with increasing accessibility to—and decreasing costs of—MRI, this advantage may become increasingly relevant in future clinical practice. Nevertheless, from a clinical standpoint, CT remains the primary modality for preoperative planning in elderly patients with cuff tear arthropathy. Importantly, in the typical elderly cuff tear arthropathy population, where reverse shoulder arthroplasty is the predominant treatment strategy, CT remains the most clinically relevant and practical modality for implant planning and navigation. In this population, the current value of ZTE MRI should be viewed as adjunctive rather than decision-changing with respect to surgical strategy. The key strength of the present study is that it is the first investigation to explore the reliability of ZTE MRI exclusively in patients with RCTA for shoulder osseous measurements, which were incorporated with correlations between the cross-sectional areas of rotator cuff muscles, 3D glenoid osseous changes, shoulder range of motion, and function.

## 5. Conclusions

ZTE MRI appears to be a promising tool for evaluating the osseous anatomy of the shoulder, particularly for angular measurements; however, assessments such as glenoid vault depth and bone loss that require 3D reconstructions still present notable technical limitations. Our findings suggest that reductions in subscapularis muscle cross-sectional area and anterior glenoid bone loss may be associated with lower functional scores and decreased active flexion and abduction in patients with RCTA. Nevertheless, these associations should not be interpreted as causal or used to derive clinical recommendations, and they warrant confirmation in larger, comparative studies.

## Figures and Tables

**Table 1:** Intraobserver and interobserver agreements for bone morphological measurements.

Measurement	Intraobserver Agreement			Interobserver Agreement		
	ICC	%95 CI	*p*	ICC	%95 CI	*p*
Glenoid version	0.906	0.826–0.949	>0.05	0.906	0.811–0.951	>0.05
Glenoid vault depth	0.905	0.824–0.948	>0.05	0.705	0.497–0.833	>0.05
Humeral subluxation index	0.877	0.773–0.933	>0.05	0.846	0.745–0.912	>0.05
Humerus head medialization	0.893	0.507–0.976	>0.05	0.857	0.573–0.965	>0.05
Anterior bone loss	0.917	0.776–0.969	>0.05	0.670	0.235–0.876	>0.05
Central bone loss	0.949	0.862–0.981	>0.05	0.788	0.525–0.919	>0.05
Posterior bone loss	0.925	0.748–0.974	>0.05	0.802	0.551–0.924	>0.05
Critical shoulder angle	0.914	0.838–0.954	>0.05	0.945	0.845–0.947	>0.05
Best-fit circle width	0.863	0.746–0.926	>0.05	0.828	0.619–0.916	>0.05
Glenoid best-fit circle bone loss ratio	0.818	0.546–0.927	>0.05	0.640	0.292–0.836	>0.05

ICC, intraclass coefficient; CI, confidence interval.

**Table 2:** Correlations between bone morphological measurements, muscle cross-sectional areas, SSc to IS + TM ratio, shoulder functional tests, and active shoulder ROMs.

Parameter		Constant–Murley Score	ASES Score	Forward Elevation	Abduction	External Rotation	Internal Rotation
Glenoid version	r	−0.118	−0.205	−0.164	−0.258	−0.123	−0.280
	*p*	0.452 ^a^	0.187 ^a^	0.294 ^a^	0.095 ^a^	0.432 ^a^	0.069 ^a^
Glenoid vault depth	r	0.096	0.263	0.133	0.108	0.082	0.047
	*p*	0.542 ^b^	0.089 ^b^	0.396 ^a^	0.493 ^a^	0.600 ^a^	0.765 ^a^
Humeral subluxation index	r	0.099	0.055	−0.028	−0.074	−0.068	−0.037
	*p*	0.529 ^a^	0.726 ^a^	0.859 ^a^	0.637 ^a^	0.664 ^a^	0.812 ^a^
Humeral head medialization	r	−0.175	−0.238	−0.274	−0.314 *	0.185	−0.194
	*p*	0.262 ^a^	0.124 ^a^	0.076 ^a^	0.040 ^a^	0.234 ^a^	0.213 ^a^
Anterior bone loss	r	−0.322 *	−0.327 *	−0.411 *	−0.475 *	−0.040	−0.277
	*p*	0.035 ^a^	0.032 ^a^	0.006 ^a^	0.001 ^a^	0.801 ^a^	0.072 ^a^
Central bone loss	r	−0.170	−0.194	−0.268	−0.354 *	0.018	−0.225
	*p*	0.277 ^a^	0.213 ^a^	0.082 ^a^	0.020 ^a^	0.910 ^a^	0.147 ^a^
Posterior bone loss	r	−0.177	−0.184	−0.259	−0.354 *	−0.009	−0.271
	*p*	0.258 ^a^	0.237 ^a^	0.093 ^a^	0.020 ^a^	0.953 ^a^	0.079 ^a^
Critical shoulder angle	r	−0.275	−0.163	−0.140	−0.155	−0.026	−0.182
	*p*	0.074 ^a^	0.296 ^a^	0.369 ^a^	0.321 ^a^	0.869 ^a^	0.242 ^a^
Best-fit circle bone loss ratio	r	0.005	−0.066	−0.043	−0.100	0.040	−0.097
	*p*	0.972 ^a^	0.673 ^a^	0.785 ^a^	0.522 ^a^	0.798 ^a^	0.535 ^a^
SS cross-sectional area	r	0.228	0.284	0.198	0.238	0.081	0.207
	*p*	0.141 ^a^	0.065 ^a^	0.203 ^b^	0.125 ^b^	0.606 ^b^	0.183 ^b^
SSc cross-sectional area	r	0.495 *	0.460 *	0.471 *	0.447 *	0.240	0.464 *
	*p*	0.001 ^b^	0.002 ^b^	0.001 ^b^	0.003 ^b^	0.121 ^b^	0.002 ^b^
IS + TM cross-sectional area	r	0.295	0.274	0.209 ^b^	0.243	0.155	0.211
	*p*	0.055 ^b^	0.075 ^b^	0.178 ^b^	0.116 ^b^	0.322 ^b^	0.173 ^b^
SSc/IS + TM ratio	r	0.180	0.175	0.276 ^b^	0.192	0.036	0.227
	*p*	0.249 ^b^	0.261 ^b^	0.074 ^b^	0.218 ^b^	0.820 ^b^	0.144 ^b^

SS, supraspinatus; SSc, subscapularis; IS, infraspinatus; TM, teres minor; ASES, American Shoulder and Elbow Surgeons; r, correlation coefficient. ^a^ Pearson correlation test; ^b^ Spearman correlation test; * significant difference present.

**Table 3:** Comparison of muscle areas, subscapularis/infraspinatus-teres minor ratio, bony morphology functional scores, and ROM values in the pseudoparesis and nonparesis groups.

Parameter	Pseudoparesis Group (*n* = 27)	Nonparesis Group (*n* = 16)	*p*
SS cross-sectional area, cm^2^	2.92 ± 0.73	3.42 ± 1.16	0.089 ^a^
SSc cross-sectional area, cm^2^	9.65 ± 2.93	11.78 ± 2.83	0.006 ^b^
IS + TM cross-sectional area, cm^2^	8.98 ± 2.54	10.17 ± 2.59	0.090 ^b^
SSc/(IS + TM) ratio	1.08 ± 0.18	1.19 ± 0.28	0.258 ^b^
Glenoid version, degrees (°)	6.04 ± 5.28	5.50 ± 5.75	0.562 ^b^
Glenoid vault depth, cm	2.08 ± 0.18	2.19 ± 0.31	0.131 ^b^
Humeral subluxation index	0.52 ± 0.06	0.53 ± 0.07	0.970 ^b^
Humerus head medialization, mm	7.04 ± 13.54	5.06 ± 17.09	0.323 ^b^
Anterior bone loss, mm	11.59 ± 14.40	4.25 ± 10.66	0.079 ^b^
Central bone loss, mm	18.29 ± 24.41	13.44 ± 21.53	0.556 ^b^
Posterior bone loss, mm	16.70 ± 22.83	13.56 ± 23.70	0.664 ^b^
Critical shoulder angle, degrees (°)	33.44 ± 4.68	31.60 ± 4.71	0.392 ^b^
Glenoid best-fit circle bone loss ratio	4.74 ± 5.27	6.02 ± 7.56	0.728 ^b^
Constant–Murley score	32.85 ± 10.53	55.75 ± 6.76	0.154 ^a^
ASES score	34.22 ± 7.96	57.12 ± 7.12	0.606 ^a^
Forward elevation, degrees (°)	60.63 ± 13.97	100.94 ± 6.64	<0.001 ^b^
Abduction degrees (°)	59.52 ± 13.07	98.12 ± 6.02	<0.001 ^b^
External rotation, degrees (°)	54.44 ± 16.25	74.37 ± 6.55	<0.001 ^b^
Internal rotation, degrees (°)	44 ± 16.45	63.37 ± 10.75	<0.001 ^b^

Data given as mean ± SD; SS, supraspinatus; SSc, subscapularis; IS, infraspinatus; TM, teres minor; ASES, American Shoulder and Elbow Surgeons; ^a^ independent sample *t*-test; ^b^ Mann–Whitney U test.

**Table 4:** Intraobserver and interobserver agreement of measurements in the current study and agreement values in similar studies that utilized CT in the literature.

	Intraobserver Agreement (ICC)		Interobserver Agreement (ICC)	
Parameter	Current Study	with CT in the Literature	Current Study	with CT in the Literature
Glenoid Version	0.906	0.605 [46]–0.99 [28]	0.906	0.456 [46] –0.915 [47]
Glenoid Vault Depth	0.905	0.97 [28]–0.976 [43]	0.877	0.19 [42]–0.58 [28]–0.866 [43]
Humeral Subluxation Index	0.846	0.841 [48]–0.966 [49]	0.705	0.639 [50]–0.94 [51]
Humeral Head Medialization	0.893	0.99 [28]	0.857	0.78 [28]
Anterior Bone Loss	0.917	0.99 [28]	0.67	0.78 [28]
Central Bone Loss	0.949	0.99 [28]	0.788	0.69 [28]
Posterior Bone Loss	0.925	0.99 [28]	0.802	0.78 [28]
Critical Shoulder Angle	0.914	0.93 [52] –0.965 [44]	0.945	0.943 [44] –0.989 [45]
Best-fit Circle Width	0.863	0.99 [53]	0.828	0.98 [53]
Best-fit Circle Bone Loss Ratio	0.818	0.90 [54] 0.95 [55]	0.64	0.74 [54] –0.98 [55]

ICC, intraclass coefficient; CT, computed tomography.

## Data Availability

The datasets used and/or analysed during the current study are available from the corresponding author upon reasonable request.
